# Supplementary material for: Sexual Dimorphism in the Skeletal Morphology of Asian Elephants (Elephas maximus): A Preliminary Morphometric Study of Skull, Scapula, and Pelvis
Source: Biology (Basel). 2025 Jul 24;14(8):933. doi: 10.3390/biology14080933 (PMC12383435; doi:10.3390/biology14080933)
Supplement: Supplementary file 1 [file biology-14-00933-s001.zip › biology-3720525-supplementary.pdf]

Supplementary data

S1: Skull measurements

Measurement		Minimum	Maximum	95% Confidence interval for mean	Mean ± Std.
FL	Female	40.5	44.5	40.4 - 44.68	42.54 ± 1.73
	Male	40.5	52.5	-29.76 - 122.76	46.5 ± 8.49
	Unknown	38.5	39.5	32.64 - 45.36	39 ± 0.71
PML	Female	36	43	35.22 - 41.78	38.5 ± 2.65
	Male	39.5	48.5	-13.19 - 101.2	44 ± 6.36
	Unknown	29.5	29.5	NaN - NaN	29.5 ± NaN
CrRH	Female	29.4	48.2	32.9 - 52.06	42.48 ± 7.72
	Male	47.5	48.4	42.23 - 53.67	47.95 ± 0.64
	Unknown	47.3	47.5	46.13 - 48.67	47.4 ± 0.14
CrRW	Female	57.6	67.8	57.4 - 67	62.2 ± 3.87
	Male	56.2	68.5	-15.82 - 140.52	62.35 ± 8.7
	Unknown	60.2	61.1	54.93 - 66.37	60.65 ± 0.64
FW	Female	26	34	25.19 - 32.93	29.06 ± 3.12
	Male	28	28.3	26.24 - 30.06	28.15 ± 0.21
	Unknown	26	32.2	-10.3 - 68.5	29.1 ± 4.38

Measurement		Minimum	Maximum	95% Confidence interval for mean	Mean ± Std.
pOW	Female	40.6	56.5	40.62 - 58.22	49.42 ± 7.09
	Male	48	57	-4.69 - 109.7	52.5 ± 6.36
	Unknown	47	54	6.02 - 94.99	50.5 ± 4.95
NW	Female	24	33	23.85 - 33.15	28.5 ± 3.74
	Male	27.5	35.5	-19.34 - 82.34	31.5 ± 5.66
	Unknown	25.5	31.5	-9.63 - 66.63	28.5 ± 4.24
pPMW	Female	22	27	22.94 - 28.06	25.5 ± 2.06
	Male	27.5	36.5	-25.19 - 89.2	32 ± 6.36
	Unknown	25	30	-4.27 - 59.28	27.5 ± 3.54
dPMW	Female	25.5	35	25.65 - 34.75	30.2 ± 3.67
	Male	35.5	42	-2.56 - 80.06	38.75 ± 4.6
	Unknown	Infinity	-Infinity	NaN - NaN	undefined ± NaN
CTS-R	Female	17	23	17.51 - 22.89	20.2 ± 2.17
	Male	24.5	35.7	-41.08 - 101.28	30.1 ± 7.92
	Unknown	Infinity	-Infinity	NaN - NaN	undefined ± NaN
CTS-L	Female	17	23	17.51 - 22.89	20.2 ± 2.17
	Male	24.5	35.7	-41.08 - 101.28	30.1 ± 7.92
	Unknown	Infinity	-Infinity	NaN - NaN	undefined ± NaN
CBL	Female	70	76.8	69.28 - 76.72	73 ± 3

Measurement		Minimum	Maximum	95% Confidence interval for mean	Mean ± Std.
	Male	76	83.7	30.92 - 128.78	79.85 ± 5.44
	Unknown	71.3	71.3	NaN - NaN	71.3 ± NaN
CZL	Female	51.5	60.5	52.78 - 62.22	57.5 ± 3.8
	Male	57.8	59.8	46.09 - 71.51	58.8 ± 1.41
	Unknown	54.2	54.5	52.44 - 56.26	54.35 ± 0.21
ZW	Female	55	63.6	54.9 - 63.14	59.02 ± 3.32
	Male	61	70.3	6.55 - 124.75	65.65 ± 6.58
	Unknown	63.3	64.4	56.86 - 70.84	63.85 ± 0.78

S2: Right Scapular measurements

		Minimum	Maximum	95% Confidence interval for mean	Mean ± Std.
DBL-R	Female	50	60	51.12 - 60.68	55.9 ± 3.85
	Male	62	72	57.43 - 72.57	65 ± 4.76
CrBL-R	Female	59	67	60.32 - 68.48	64.4 ± 3.29
	Male	69	73	67.33 - 74.67	71 ± 2.31
CaBL-R	Female	34	43	33.02 - 41.98	37.5 ± 3.61
	Male	36	43	34.91 - 44.09	39.5 ± 2.89
MLD-R	Female	16	21	14.97 - 21.53	18.25 ± 2.06

		Minimum	Maximum	95% Confidence interval for mean	Mean ± Std.
	Male	18	27	14.08 - 27.42	20.75 ± 4.19
MLV-R	Female	41	57	39.59 - 60.91	50.25 ± 6.7
	Male	51.5	56	47.81 - 59.19	53.5 ± 2.29
LS-R	Female	7	8	6.92 - 8.28	7.6 ± 0.55
	Male	8	9	7.95 - 9.55	8.75 ± 0.5
LGC-R	Female	14	18	13.98 - 18.42	16.2 ± 1.79
	Male	15	18	14.15 - 18.35	16.25 ± 1.32
WSN-R	Female	17	19	16.76 - 18.84	17.8 ± 0.84
	Male	16	20	15.25 - 20.5	17.88 ± 1.65
WSH-R	Female	19	21	19.16 - 21.24	20.2 ± 0.84
	Male	20	23	18.75 - 23.25	21 ± 1.41
LSS-R	Female	47	54	46.34 - 55.91	51.13 ± 3.01
	Male	50	67	46.32 - 69.18	57.75 ± 7.18
LSP-R	Female	15.5	21	15.07 - 22.68	18.88 ± 2.39
	Male	15	23	13.49 - 24.01	18.75 ± 3.3

S3: Left Scapular measurements

		Minimum	Maximum	95% Confidence interval for mean	Mean ± Std.
DBL-L	Female	56	61	57.12 - 60.88	59 ± 1.79
	Male	62	67	57.43 - 70.57	64 ± 2.65
CrBL-L	Female	57	68	59.55 - 68.45	64 ± 4.24

		Minimum	Maximum	95% Confidence interval for mean	Mean ± Std.
	Male	68	74	63.74 - 78.92	71.33 ± 3.06
CaBL-L	Female	34	39	34.92 - 39.41	37.17 ± 2.14
	Male	39	44	34.79 - 47.54	41.17 ± 2.57
MLD-L	Female	18	24	18.05 - 23.12	20.58 ± 2.42
	Male	17	22	13.41 - 25.92	19.67 ± 2.52
MLV-L	Female	41	55	42.48 - 53.35	47.92 ± 5.18
	Male	54	55	53.23 - 56.1	54.67 ± 0.58
LS-L	Female	7	8	6.79 - 7.88	7.33 ± 0.52
	Male	9	10	7.9 - 10.77	9.33 ± 0.58
LGC-L	Female	13.5	16	13.61 - 15.89	14.75 ± 1.08
	Male	15	19	11.5 - 21.84	16.67 ± 2.08
WSN-L	Female	13	18	13.98 - 18.02	16 ± 1.92
	Male	17	20	13.7 - 22.3	18 ± 1.73
WSH-L	Female	17	21	17.99 - 20.84	19.42 ± 1.36
	Male	19	24	15.41 - 27.92	21.67 ± 2.52
LSS-L	Female	45	58	46.56 - 56.1	51.33 ± 4.55
	Male	49.5	67	34.93 - 79.4	57.17 ± 8.95
LSP-L	Female	14	20	12.61 - 21.64	17.13 ± 2.84
	Male	16	20	12.5 - 22.84	17.67 ± 2.08

S4: Pelvic measurements

		Minimum	Maximum	95% Confidence interval for mean	Mean ± Std.
HWPG	Female	99.5	105	100.64 - 103.86	102.25 ± 1.93
	Male	103	114	94.17 - 121.83	108 ± 5.57
HWPA	Female	36	41	36.87 - 39.63	38.25 ± 1.65
	Male	37	38	35.9 - 38.77	37.33 ± 0.58
HWA	Female	30	33	30.18 - 32.07	31.13 ± 1.13
	Male	31	34	27.7 - 36.3	32 ± 1.73
WEI	Female	51.5	61	53.25 - 58.02	55.64 ± 2.86
	Male	54	64	46.39 - 71.28	58.83 ± 5.01
LPG	Female	67	85	74.05 - 83.57	78.81 ± 5.69
	Male	84	93	76.62 - 99.38	88 ± 4.58
LPS	Female	26	32.5	28.12 - 32.26	30.19 ± 2.48
	Male	32.5	37	29.3 - 41.04	35.17 ± 2.36
DHPA	Female	27.5	52	36.24 - 49.76	43 ± 8.08
	Male	38	51.5	27.21 - 61.12	44.17 ± 6.83
WIW	Female	33	48.5	35.7 - 42.92	39.31 ± 4.32
	Male	41	45	37.5 - 47.84	42.67 ± 2.08
LIl	Female	60	68	61.74 - 66.63	64.19 ± 2.93
	Male	63	68	59.08 - 71.59	65.33 ± 2.52
WIS	Female	12	16	13.5 - 15.5	14.5 ± 1.2
	Male	12	16	9.03 - 18.97	14 ± 2
PIl	Female	36.5	41	36.97 - 39.28	38.13 ± 1.38
	Male	38	50	27.49 - 58.51	43 ± 6.24

		Minimum	Maximum	95% Confidence interval for mean	Mean ± Std.
PPS	Female	13	17	13.75 - 15.98	14.86 ± 1.33
	Male	16	25	7.08 - 31.59	19.33 ± 4.93
PIs	Female	18	21	19.13 - 20.95	20.04 ± 1.09
	Male	19.5	25	15.33 - 29.01	22.17 ± 2.75
LFO	Female	12	15	12.13 - 13.87	13 ± 1.04
	Male	13	14	12.23 - 15.1	13.67 ± 0.58
LA	Female	11	13	11.79 - 12.96	12.38 ± 0.69
	Male	13	15	10.8 - 16.54	13.67 ± 1.15
HAT	Female	24.5	34	26.05 - 31.45	28.75 ± 3.23
	Male	29	33	24.6 - 36.07	30.33 ± 2.31
